# Can We Identify or Exclude Extensive Axillary Nodal Involvement in Breast Cancer Patients Preoperatively?

**DOI:** 10.1155/2019/8404035

**Published:** 2019-11-22

**Authors:** Martijn Leenders, Gaëlle Kramer, Kamar Belghazi, Katya Duvivier, Petrousjka van den Tol, Hermien Schreurs

**Affiliations:** ^1^Department of Surgery, Northwest Clinics, Wilhelminalaan 12, 1815 JD Alkmaar, Netherlands; ^2^Department of Radiology, VU Medical Centre, De Boelelaan 1117, 1081 HV Amsterdam, Netherlands; ^3^Department of Surgery, VU Medical Centre, De Boelelaan 1117, 1081 HV Amsterdam, Netherlands

## Abstract

**Background:**

Breast cancer treatment has rapidly changed in the last few years. Particularly, treatment of patients with axillary nodal involvement has evolved after publication of several randomized clinical trials. Omitting axillary lymph node dissection in selected early breast cancer patients with one or two positive sentinel nodes did not compromise overall survival nor regional disease control in these trials. Hence, either excluding or identifying extensive axillary nodal involvement becomes increasingly important.

**Purpose:**

To evaluate whether the current diagnostic modalities can accurately identify or exclude extensive axillary nodal involvement. Evaluated modalities were axillary ultrasound, ultrasound-guided needle biopsy, MRI, and PET/CT.

**Methods:**

A literature search was performed in the Cochrane Library, EMBASE, and PubMed databases up to June 2019. The search strategy included terms for breast cancer, lymph nodes, and the different imaging modalities. Only articles that reported pathological *N*-stage or the total number of positive axillary lymph nodes were considered for inclusion. Studies with patients undergoing neoadjuvant systemic therapy were excluded.

**Conclusion:**

There is no evidence that any of the current preoperative axillary imaging modalities can accurately exclude or identify breast cancer patients with extensive nodal involvement. Both negative PET/CT and negative MRI scans (with gadolinium-based contrast agents) are promising in excluding extensive nodal involvement. Larger studies should be performed to strengthen this conclusion. False-negative rates of axillary ultrasound and ultrasound-guided needle biopsy are too high to rely on negative results of these modalities in excluding extensive nodal involvement.

## 1. Introduction

Since the first radical mastectomy by Halsted in the late 1800s, breast cancer surgery has become much less mutilating. Currently, results of breast-conserving surgery are excellent, due to early detection by breast cancer screening programs and the application of (neo)adjuvant treatment modalities. Axillary treatment is rapidly evolving as well. The results of the ACOSOG Z0011 [1], IBCSG 23-01 [2], and AATRM 048/13/2000 [3] trials demonstrated that selected breast cancer patients with one or two positive sentinel nodes do not benefit from completion ALND (cALND) in terms of local control, disease-free survival, and overall survival [1–3]. In these trials, only clinical T1-2N0 patients undergoing breast-conserving surgery followed by whole breast irradiation and systemic therapy were included. Therefore, most national guidelines now recommend avoiding cALND in breast cancer patients with one or two positive sentinel nodes who meet these eligibility criteria. Hence, the outcome of the sentinel node biopsy (SNB) in this subgroup of patients has actually no clinical consequences. Consequently, randomized clinical trials are recently initiated to study whether SNB can be safely omitted in these patients (SOUND [4], BOOG 2013-08 [5], and INSEMA). However, the presence of extensive nodal involvement should still be excluded. Patients with extensive nodal involvement do not meet the eligibility criteria of the ACOSOG Z0011 trial and should therefore receive axillary treatment according to the recent guidelines. Ideally, this presence of extensive nodal involvement is accurately excluded by negative results of preoperative axillary imaging modalities. Patients with extensive axillary nodal involvement are at a much higher risk for locoregional recurrence. Irradiation of regional lymph nodes is therefore advised in these patients since it improves prognosis [6–8]. Identifying extensive nodal involvement is thus an important selection factor for postoperative regional radiotherapy. Neoadjuvant systemic therapy is increasingly implemented in patients with node-positive disease. Hence, the number of positive nodes is preferably estimated by a noninvasive axillary imaging modality before the start of neoadjuvant systemic therapy.

The first aim of this review was to evaluate whether negative results of the current preoperative axillary imaging modalities can exclude extensive nodal involvement in newly diagnosed breast cancer patients. Modalities that were evaluated are axillary ultrasound (AxUS), ultrasound-guided needle biopsy (UNB), magnetic resonance imaging (MRI), and fluorine-18-fluoro-2-deoxy-D-glucose positron emission tomography/computed tomography (PET/CT). The second aim of this review was to evaluate whether these diagnostic modalities can distinguish breast cancer patients with extensive axillary nodal involvement from patients with no or limited nodal involvement.

## 2. Methods

A literature search was performed in the Cochrane Library, EMBASE, and PubMed databases up to June 2019. Filters used were English language and human studies. The search strategy included terms for breast cancer, lymph nodes, and the different diagnostic entities (AxUS, UNB, MRI, and PET/CT). The inclusion criteria were as follows: (1) newly diagnosed, histologically proven breast cancer patients, (2) the diagnostic modality was performed preoperatively, (3) node histology (based on SNB and/or ALND) was the reference standard for ascertaining presence/absence of metastatic nodes, (4) cALND was performed in case of a positive sentinel node, and (5) pathological *N*-stage or total number of positive nodes was reported. Exclusion criteria were (1) studies with patients undergoing neoadjuvant systemic therapy.

To determine whether negative results of preoperative axillary imaging modalities can exclude extensive nodal involvement, both negative predictive values (NPVs) and false-negative rates (FNRs) were calculated from the raw data of all included studies. For this purpose, NPV is defined as the proportion of patients with a negative test result that does not have extensive nodal involvement histologically (i.e., number of patients with a negative test result who did not have extensive nodal involvement histologically/number of patients with a negative test result). FNR is defined as the proportion of patients with extensive nodal involvement which yielded a negative test result (i.e., number of patients with extensive nodal involvement and a negative test result/number of patients with extensive nodal involvement). The combination of NPV and FNR was used to determine which diagnostic entity is the optimal test to exclude extensive nodal involvement.

To evaluate whether preoperative axillary imaging modalities can accurately distinguish patients with no or limited axillary nodal involvement from patients with extensive nodal involvement, we evaluated studies that were specifically designed for this purpose.

Currently, there is some ambiguity on the definition of extensive nodal involvement. The ACOSOG Z0011 trial defined extensive nodal involvement as ≥3 positive nodes. However, according to TNM classification, extensive nodal involvement is defined as ≥4 histologically positive nodes (pN2-3). In this review, we will quantify the number of positive nodes whenever needed to avoid ambiguity.

## 3. Results

### 3.1. Axillary Ultrasonography

#### 3.1.1. Excluding Extensive Nodal Involvement

A number of studies on accuracy of AxUS described the total number of histologically positive nodes. In Table 1, all studies that included at least 200 patients are summarized. In studies that defined extensive nodal involvement as pN2-3 (that is ≥ 4 positive nodes), the FNR ranges from 10 to 50% (mean 18%). Thus, on average, 18% of patients with extensive nodal involvement had a normal AxUS. The NPV of a normal AxUS to exclude extensive nodal involvement ranges from 92 to 97% (mean 96%) in these studies [9–15]. In studies that defined extensive nodal involvement as ≥3 positive nodes, the FNR ranges from 30 to 37% (mean 34%). NPV in these studies ranges from 92 to 98% (mean 95%) (Table 1) [16–23].

#### 3.1.2. Identifying Patients with Extensive Nodal Involvement

In the post-ACOSOG Z0011 era, several studies examined whether AxUS could be used to distinguish between limited and extensive axillary nodal involvement. Most studies focused on the association between the number of sonographically suspicious nodes and the extent of (pathological) nodal disease. These studies concluded that the mean number of positive axillary nodes is significantly higher amongst patients with their positive nodes identified by AxUS than by SNB [14, 19, 24]. Further, the presence of extensive nodal involvement is significantly associated with the number of sonographically suspicious nodes in these studies [12, 17, 18, 21, 25–31]. For instance, Kim et al. [18] showed that the percentage of patients with ≥3 positive nodes was 3.1% vs. 38.5% vs. 62.5% in case of ≤1, 2, or ≥3 sonographically suspicious nodes (*P* < 0.001). In multivariate analysis, the odds ratios for ≥3 positive nodes were significantly increased when two suspicious nodes were seen (OR 6.52, 95% CI 1.36–31.28) and when more than two suspicious nodes were seen (OR 21.08, 95% CI 2.57–172.86) [18]. Farrell et al. [25] showed that the mean number of histologically positive nodes was significantly higher if more than two suspicious nodes were detected sonographically compared to two or only one (10.1 vs. 7.5 vs. 5.2) [25]. Studies differentiating between cN0, cN1, and cN2-3 disease (based on sonographic number of suspicious axillary nodes) also showed that the percentage of patients with pN2-3 disease was significantly higher in case of cN1 or cN2-3 disease compared to cN0 disease [12, 26, 27]. In patients who were sonographically classified as cN2-3, the percentage of patients with pN2-3 disease was very high, that is, 100% (2 of 2) [27], 100% (2 of 2) [26], and 84% (46 of 55) [12], respectively. However, when using this threshold of cN2-3, a lot of patients with pN2-3 were missed since the FNR was 87.5% [27], 95.7% [26], and 31.3% [12], respectively. In short, the extent of nodal involvement is significantly associated with the number of sonographically suspicious nodes. The most optimal cutoff to distinguish between limited and extensive nodal involvement has not been determined yet.

Several studies examined the association between maximum cortical thickness of the most suspicious node and the extent of nodal involvement. All studies concluded that increased cortical thickness was an independent predictor of extensive nodal involvement [10, 18, 28, 32, 33]. The most optimal cutoff value to discriminate between limited and extensive nodal involvement ranges between 3.0 and 5.0 millimeters in these studies [10, 18, 28]. For instance, Lim et al. [33] used a cutoff point of maximum cortical thickness of 4.0 millimeters. When maximum cortical thickness was larger than 4.0 millimeters, PPV to predict ≥3 positive nodes was 82.5% (33/40). However, the FNR at this cutoff was 68.6% (72/105). Hence, the majority of patients with ≥3 positive nodes were missed when using this cutoff point [33].

One study used a combination of morphology of suspicious nodes and number of sonographically suspicious nodes to predict pN2-3 disease [9]. In this study, one experienced radiologist retrospectively reviewed AxUS images of 559 patients and determined whether there were any suspicious nodes. The suspicious nodes were categorized with a grade of suspicion of high (complete or near-complete absence of fatty hilum), medium (cortical thickness >4 mm or asymmetrical cortical thickening >3 mm), or low (uniform cortical thickening of 3-4 mm). They reported that pN2-3 disease was highly likely (PPV of 82%) when there were at least two lymph nodes of high suspicion. By using this cutoff, many patients with pN2-3 disease were missed because sensitivity of predicting pN2-3 disease was only 54% [9].

### 3.2. Ultrasound-Guided Needle Biopsy

#### 3.2.1. Excluding Extensive Nodal Involvement

In Table 2, we listed all studies on accuracy of AxUS followed by ultrasound-guided needle biopsy of a suspicious node (AxUS/UNB) that provided data on the definite number of histologically positive nodes. Only two studies primarily performed ultrasound-guided core needle biopsy (CNB) [26, 27]. Both studies defined extensive nodal involvement as pN2-3 disease (Table 2). All studies that defined extensive nodal involvement as ≥3 positive nodes performed ultrasound-guided fine-needle aspiration cytology (FNAC). In these studies, the FNR of AxUS/FNAC ranges from 15 to 58% (mean 43%). Thus, on average, 43% of patients with ≥3 positive nodes had a negative AxUS/FNAC (either AxUS− or AxUS+/FNAC−). The NPV of a negative AxUS/FNAC to exclude ≥3 positive nodes ranges from 93 to 97% (mean 95%). Hence, the chance of ≥3 positive nodes is approximately 5% in case of a negative AxUS/FNAC (Table 2) [16, 17, 19, 25, 28, 35].

#### 3.2.2. Identifying Patients with Extensive Nodal Involvement

Many studies evaluated the role of US/UNB in selecting patients with extensive nodal disease. Most of these studies evaluated axillary tumor burden amongst different breast cancer patient subgroups (UNB-positive versus SNB-positive patients). Recently, three meta-analyses on this subject were published [36–38]. Houssami and Turner [37] estimated that, amongst patients with positive nodes, the odds ratio for high nodal disease burden (≥4 positive nodes) was 4.38 (95% CI 3.13–6.13) for a positive UNB versus a negative UNB [37]. Wely van et al. [36] compared the number of patients with histological N1 and N2-3 disease between patients with a positive UNB (UNB+) and patients with a negative UNB but positive SNB (UNB−/SNB+). The preferred method of biopsy was FNAC in about 90% of patients. Significantly more patients in the UNB+ group had pN2-3 disease than in the UNB−/SNB+ group (*P* < 0.001), that is, 56.0% (298 of 532) versus 23.8% (59 of 248) [36]. Ahmed et al. [38] compared the number of patients with ≤2 metastatic nodes and ≥3 metastatic nodes, in addition to only considering macrometastases. They concluded that significantly more patients in the UNB+ group had ≥3 metastatic nodes compared to the SNB+ group (odds ratio 5.95, 95% CI 5.80–6.11). The mean number of macrometastatically involved nodes was 2.9 (standard error 0.2) for the UNB+ and 1.6 (standard error 0.2) for the SNB+ group. Finally, cumulative probability identified that 56.8% of UNB+ patients and 21.1% of SNB+ patients had ≥3 macrometastatic nodes [38]. In short, the mean number of positive nodes is significantly higher in UNB+ patients compared to SNB-positive patients. Approximately 56% of patients with their positive nodes identified by UNB will ultimately have extensive nodal involvement.

Ideally, these UNB+ patients with extensive nodal involvement can be distinguished from UNB+ patients with limited nodal involvement preoperatively. A few recent studies addressed this topic and compared clinical, radiological, and pathological features between these two UNB+ subgroups [30, 33, 39]. Features that were significantly associated with having extensive nodal involvement at final pathology in UNB+ patients were as follows: larger tumor size [30, 39], lobular histology [30, 39], a higher grade of lymphovascular invasion [39], maximum cortical thickness of the most suspicious node larger than 4.0 millimeters [33], and having more than one suspicious lymph node on AxUS [30, 33, 39]. For UNB+ patients with both primary tumor size on imaging ≤2 centimeters and one abnormal node on AxUS, only 27% had N2-3 disease at final pathology (*P*=0.007) [30].

### 3.3. Magnetic Resonance Imaging

#### 3.3.1. Excluding Extensive Nodal Involvement

In total, six studies evaluating the accuracy of MRI provided data on the total number of (histologically) positive nodes (Table 3). All studies used a MRI system with a coil covering breast and axilla. Three studies defined extensive nodal involvement as ≥3 positive nodes. Two of these three studies performed dynamic contrast-enhanced MRI (DCE-MRI) with gadolinium-based contrast agents [17, 18]. One of these studies performed unenhanced MRI [16]. Accuracy of DCE-MRI to exclude ≥3 positive nodes seems to be better than unenhanced MRI. The FNR of DCE-MRI ranged from 12 to 19% (mean 17%) and NPV ranged from 97 to 99% (mean 98%) (Table 3) [17, 18].

#### 3.3.2. Identifying Patients with Extensive Nodal Involvement

Only few studies examined whether MRI could be used to distinguish between limited and extensive nodal involvement. Kim et al. [18] compared clinical, radiological, and pathological features between early breast cancer patients with ≤2 positive nodes and patients with ≥3 positive nodes. They performed DCE-MRI with gadolinium-based contrast agents (field strength of 3.0 T). In multivariate analysis, a higher number of suspicious lymph nodes on MRI was significantly associated with the presence of ≥3 positive nodes on final pathology (2 suspicious nodes, OR 69.0, *P*=0.001; ≥3 suspicious nodes, OR = 93.55, *P* < 0.001). In patients with no or only one suspicious node on MRI, the percentage of patients with ≥3 positive nodes on final pathology was 1.8% (4 of 225). In contrast, in patients with two or more suspicious nodes on MRI, the percentage of patients with ≥3 positive nodes on final pathology was 41.9% (13 of 31) (*P* < 0.001). When using this threshold of two suspicious nodes on MRI, 4 of 17 (23.5%) patients with ≥3 positive nodes on final pathology would have been missed. Cortical morphologic changes of the most suspicious node on MRI were not independently associated with ≥3 positive nodes on final pathology in the series of Kim et al. [18]. Hieken et al. [17] performed a similar study. They differentiated between no, only one, and more than one suspicious lymph node on magnetic resonance images. Images were obtained by DCE-MRI (1.5 T) using gadolinium in 505 breast cancer patients. If no, only one, or more than one suspicious node was seen on MRI, the percentage of patients with ≥3 positive nodes on final pathology was 3.0% (10 of 337), 15.1% (11 of 73), and 32.6% (31 of 95), respectively (*P*=0.008) [17]. When using a threshold of two suspicious nodes on MRI, 32.6% of patients had ≥3 positive nodes on final pathology. At this threshold, 40.4% (21 of 52) of patients with ≥3 positive nodes on final pathology would have been missed. Next, Hyun et al. [41] performed DCE-MRI (3.0 T) using gadolinium in 310 breast cancer patients. They differentiated between cN0, cN1, and cN2-3 based on MRI. If no abnormal nodes were identified (cN0), 0.4% (1 of 257) was ultimately shown to have pN2-3 disease by histopathology. 45 cases were staged as cN1, of which 7 were pN2-3 (15.6%). Finally, 8 cases were staged as cN2-3, of which 4 were pN2-3 (50%) [41]. A similar study was performed by Nijnatten van et al. [27]. They evaluated the axillary regions of 377 breast cancer patients on unenhanced T2-weighted sequences. Their results were virtually identical to the results of Hyun et al. [41].

At last, two studies analyzed if MRI can be used to distinguish UNB+ patients with extensive nodal involvement from UNB+ patients with limited nodal involvement. Hieken et al. [17] concluded that the chance of ≥3 positive nodes was significantly higher in UNB+ patients with more than one suspicious node on MRI compared to patients with only one suspicious node on MRI (67.6% vs. 30.3%, *P*=0.005) [17]. In a study of Pilewskie et al. [39], a trend was shown that the rate of ≥3 positive nodes was higher in UNB+ patients with more than one suspicious node on MRI compared to only one or no suspicious nodes (*P*=0.083).

### 3.4. 18F-FDG PET/CT

#### 3.4.1. Excluding Extensive Nodal Involvement

The combination of metabolic and morphologic data can be obtained with integrated 18F-FDG PET/CT systems. Only two studies that performed 18F-FDG PET/CT reported the total number of histologically positive nodes (Table 4). One of these studies defined extensive nodal involvement as ≥3 positive nodes [32]. In this study, 21% of patients with ≥3 positive nodes had a negative (axillary) PET/CT. The NPV of a negative (axillary) PET/CT to exclude ≥3 positive nodes was 97% in this study [32]. The study that defined extensive nodal involvement as pN2-3 had similar results: FNR 28% and NPV 98% [40].

#### 3.4.2. Identifying Patients with Extensive Nodal Involvement

Several studies were published on axillary tumor burden according to PET/CT scans in newly diagnosed breast cancer patients with *N*+ disease [6]. However, patients were treated with neoadjuvant chemotherapy in these studies. No single study on locoregional staging with PET/CT compared the number of suspicious axillary lymph nodes on PET/CT with pathological nodal burden without systemic therapy in between. Therefore, only circumstantial evidence is available to answer the question if PET/CT is helpful in predicting the extent of axillary nodal involvement.

First, the percentages of patients with extensive nodal involvement were 23.2% [40], 32.4% [32], and 64.0% [42] in three studies when PET/CT was positive for axillary nodal involvement. These newly diagnosed breast cancer patients all had clinically negative axillae.

Fuster et al. [43] performed PET/CT in sixty consecutive patients with primary breast carcinomas larger than three centimeters. Axillary lymph node metastases were confirmed in 20 of 52 patients with ALND. In these 20 patients, 84 of 315 nodes were histologically confirmed to contain metastases. On a lymph node count, PET/CT detected 24 of these 84 metastatic lymph nodes. Hence, the best lesion-based sensitivity in their study was 28.6%. Unfortunately, the authors did not differentiate between micro- and macrometastases [43]. Heusner et al. [44] performed a similar study. They analyzed FDG avidity of all lymph nodes ≥5 mm that were noted on CT. Any focus which could be mapped to a lymph node and was considered elevated above normal was rated as positive for metastasis. PET/CT results were correlated to histopathological findings of ALND. Of 61 patients, 24 had positive nodes on final histopathology (150 positive nodes in total). In 14 true-positive PET/CT scans, 60 suspicious lymph nodes were detected. When considering these 60 lymph nodes as truly positive nodes, their best lesion-based sensitivity was 41% [44]. However, no node-to-node correlation between avid nodes on PET/CT and histologically metastatic nodes was performed in these studies. Theoretically, PET-positive nodes may have been false-positive due to inflammation for instance. Thus, the true lesion-based sensitivity for 18F-FDG PET/CT may be lower than the percentages reported in these two studies. In short, 18F-FDG PET/CT highly underestimates the number of metastatic axillary lymph nodes.

## 4. Discussion

Treatment of clinical T1-2N0 breast cancer patients with one or two metastatic sentinel nodes has changed after publication of the ACOSOG Z0011 [1], IBCSG 23-01 [2], and AATRM 048/13/2000 [3] trials. Omitting completion ALND in these patients did not compromise overall survival nor regional disease control. Only patients who were treated by breast-conserving surgery, whole-breast irradiation, and adjuvant systemic therapy were included in these trials. Excluding extensive nodal involvement rather than detecting occult node-positive disease has become increasingly important since then. Ideally, this task is fulfilled by preoperative axillary imaging modalities. Therefore, we questioned if negative results of AxUS, AxUS/UNB, MRI, and PET/CT can exclude the presence of extensive nodal involvement in newly diagnosed breast cancer patients. In some studies that we reviewed, the number of patients with ≥3 positive nodes was described. In other studies, the number of patients with pN1 (1 to 3 positive nodes) and pN2-3 (≥4 positive nodes) was described. We were particularly interested in excluding the presence of ≥3 positive nodes. When the presence of ≥3 positive nodes can be excluded preoperatively in breast cancer patients that meet the ACOSOG Z0011 eligibility criteria, surgically staging the axilla (by SNB) is redundant in these patients.

When applied to exclude extensive nodal involvement, the ultimate axillary staging modality is featured by a NPV of 100% and a FNR of 0%. Only ALND approaches these requirements. Even SNB is featured by a false-negative rate of 9.8% in the largest randomized clinical trial comparing SNB with ALND in patients with clinically node-negative breast cancer [45]. Using SNB as a reference standard is therefore a drawback of this review article. In the control (cALND) arm of the ACOSOG Z0011 trial, 21.0% had ≥3 positive nodes. Despite this high rate of extensive nodal involvement, there was a very low rate of axillary recurrences in the no-ALND arm (1.6% after 5 years) [1]. This discrepancy was probably due to the effects of adjuvant systemic therapy and whole-breast irradiation, which eliminated residual axillary metastases. In our opinion, the FNRs of preoperative axillary staging methods that are applied to exclude ≥3 positive nodes (in patients who meet the ACOSOG Z0011 eligibility criteria) should not exceed this rate of 21.0%.

In Table 5, NPVs and FNRs of all preoperative axillary imaging modalities to exclude ≥3 positive nodes are summarized. NPVs of all modalities to exclude ≥3 positive nodes are very high (mean NPVs of all modalities approximately 95%). These very high NPVs can (partially) be explained by the low prevalence of ≥3 positive nodes because NPV is inversely related to prevalence. Since FNR is intrinsic to a test, the FNR might be more helpful to determine which imaging modality might best be used to exclude ≥3 positive nodes.

AxUS and AxUS/UNB are the most widely studied preoperative axillary imaging modalities. FNRs of both negative AxUS and negative AxUS/FNAC (either AxUS− or AxUS+ FNAC−) were well above the arbitrarily decided maximum FNR of 21%. Thus, when relying on negative AxUS or negative AxUS/FNAC results, too many patients with ≥3 positive nodes would be missed and would not be properly treated. This could negatively influence the number of locoregional recurrences and prognosis [6–8]. AxUS/FNAC performs worse than AxUS in excluding ≥3 positive nodes. This worse FNR of AxUS/FNAC to exclude ≥3 positive nodes can be explained by the fact that the false-negative results of FNAC are added to the false-negative results of AxUS.

Evidence regarding the diagnostic performance of both MRI and PET/CT in excluding ≥3 positive nodes is very scarce. DCE-MRI with gadolinium-based contrast agents seems to perform better in excluding ≥3 positive nodes than nonenhanced MRI albeit DCE-MRI showed less promising results in a previous systematic review on the role of MRI in axillary lymph node imaging [46]. In both studies that evaluated DCE-MRI with gadolinium-based contrast agents, the FNR of a negative MRI to exclude ≥3 positive nodes was lower than 21%. The same holds for PET/CT. Only one PET/CT study met our inclusion criteria, and in this study, the FNR of a negative PET/CT to exclude ≥3 positive nodes was 20.9%. Hence, both negative PET/CT and negative DCE-MRI (with gadolinium-based contrast agents) seem promising in excluding ≥3 positive nodes. However, this conclusion is based on only one PET/CT study and two DCE-MRI studies. Before we can rely on these diagnostic modalities in excluding ≥3 positive nodes, larger studies should be performed to confirm these results.

Our second aim was to evaluate whether these preoperative axillary imaging modalities can accurately distinguish patients with extensive nodal involvement from patients with no or only limited axillary nodal involvement. In short, none of the investigated preoperative axillary imaging modalities can. A higher number of suspicious lymph nodes on AxUS and MRI are significantly associated with extensive nodal involvement. However, the most optimal cutoff (e.g., ≥2 suspicious nodes on AxUS or MRI) to distinguish between limited and extensive nodal involvement has not been determined yet. With rising cutoff values, positive predictive values to predict extensive nodal involvement will rise, but false-negative rates will rise as well (more patients with extensive nodal involvement will be missed). Therefore, the ideal cutoff values may not exist. Mathematical models (nomograms, scores, and prediction rules) combining clinical, pathological, and radiological parameters may be more useful to estimate the chance of extensive nodal involvement in patients with suspicious nodes on AxUS or MRI and in UNB+ patients. Development of these models should be subject for future studies.

Currently, either excluding or identifying extensive nodal involvement in breast cancer patients is of great importance for reasons described above. However, it could be argued that the role of axillary staging will diminish in the near future. In the NSABP B-04 trial, clinically node-negative breast cancer patients were randomized for mastectomy, mastectomy with ALND, or mastectomy with axillary radiotherapy in the 1970s. About 40% of patients in the ALND group had pathological confirmation of tumor-positive axillary lymph nodes. However, less than half of these patients (18.6%) had clinically apparent ipsilateral nodal metastases during 25 years of follow-up. None of these patients was treated with systemic therapy or axillary radiotherapy [47]. This discrepancy between axillary recurrences in the no-ALND group and the rate of nonsentinel node metastases in the ALND group was also shown in the ACOSOG Z0011 [1] and IBCSG 23-01 trials [2]. In these latter trials, systemic therapy and radiotherapy have contributed to this discrepancy since almost all patients were treated with systemic therapy and some form of radiotherapy. Even more important, disease-free survival and overall survival were not affected by omitting ALND in any of these trials. Hence, leaving positive axillary nodes unremoved might well not be that big a problem, particularly because the majority of patients are nowadays treated with adjuvant systemic therapy that is increasingly more effective. The current FNR of 31% of a negative AxUS to exclude extensive nodal involvement might therefore be acceptable in the future. The long-term results of randomized clinical trials in which SNB is omitted and axillary staging is performed exclusively by AxUS (SOUND [4], BOOG 2013-08 [5], and INSEMA) might provide us with some answers.

## 5. Conclusion

Currently, there is no preoperative axillary imaging modality that can neither identify nor exclude breast cancer patients with extensive nodal involvement accurately. Both negative PET/CT and negative DCE-MRI scans (with gadolinium-based contrast agents) are promising in excluding ≥3 positive nodes in breast cancer patients. Larger studies should be performed to strengthen this conclusion. False-negative rates of AxUS and AxUS/FNAC are too high to rely on negative results of these modalities in excluding ≥3 positive nodes. However, with increasingly more effective (neo) adjuvant therapy, these current false-negative rates might be acceptable in the future.

## Figures and Tables

**Table 1:** Summary of studies on axillary ultrasonography to exclude patients with extensive nodal involvement.

Study	Year	No. of patients	No. (%) of patients with extensive nodal involvement	No. of false negatives	No. of true negatives	NPV (%)	FNR (%)
*Studies in which extensive nodal involvement was defined as pN2-3*							
Abe et al. [9]	2013	559	60 (10.7%)	10	368	97	17
Amonkar et al. [10]	2013	439	44 (10.0%)	10	283	97	23
Jackson et al. [11]	2015	494	48 (9.7%)	14	369	96	29
Kijima et al. [12]	2010	380	67 (17.6%)	10	238	96	15
Liu et al. [13]	2018	3944	664 (16.8%)	64	2236	97	10
Wely van et al. [14]	2013	1448	178 (12.3%)	89	1094	92	50
Zhang et al. [15]	2015	1049	207 (19.7%)	33	619	95	16
Mean						96	18

*Studies in which extensive nodal involvement was defined as ≥3 positive nodes*							
Barco et al. [16]	2016	1533	210 (13.7%)	64	1127	95	30
Hieken et al. [17]	2013	906	76 (8.4%)	23	620	96	30
Kim et al. [18]	2019	311	19 (6.1%)	6	238	98	32
Kramer et al. [19]	2016	2130	248 (11.6%)	91	1491	94	37
Lee et al. [20]	2013	210	38 (18.1%)	12	130	92	32
Lim et al. [21]	2019	1298	180 (13.9%)	62	950	94	34
Moorman et al. [22]	2014	1060	102 (9.6%)	37	842	96	36
Morrow et al. [23]	2018	4695	594 (12.7%)	206	3374	94	35
Mean						95	34

AxUS = axillary ultrasonography; no. of false negatives = number of patients with normal/negative AxUS but histologically extensive nodal involvement; no. of true negatives = number of patients with normal/negative AxUS and histologically no/limited nodal involvement; NPV = negative predictive value; FNR = false-negative rate.

**Table 2:** Summary of studies on US-guided needle biopsy to exclude patients with extensive nodal involvement.

Study	Year	No. of patients	No. (%) of patients with extensive nodal involvement	No. of false negatives	No. of true negatives	NPV (%)	FNR (%)
*Studies in which extensive nodal involvement was defined as pN2-3*							
US/FNAC							
Gipponi et al. [34]	2016	400	42 (10.5%)	15	329	96	36
Wely van et al. [14]	2013	1448	178 (12.3%)	99	1154	92	56
US/CNB							
Nijnatten van et al. [27]	2016	377	16 (4.2%)	5	339	99	31
Schipper et al. [26]	2013	577	47 (8.1%)	23	499	94	49

*Studies in which extensive nodal involvement was defined as ≥3 positive nodes*							
US/FNAC							
Barco et al. [16]	2016	1506	200 (13.3%)	74	1223	94	37
Farrell et al. [25]	2015	322	29 (9.0%)	9	281	97	31
Hieken et al. [17]	2013	906	76 (8.4%)	34	790	96	45
Kramer et al. [19]	2016	2130	248 (11.6%)	137	1802	93	55
Wallis et al. [35]	2018	769	36 (4.7%)	21	716	97	58
Zhu et al. [28]	2016	445	84 (18.9%)	13	314	96	15
Mean						95	43

US/FNAC = axillary ultrasonography followed by fine-needle aspiration cytology of suspicious nodes; US/CNB = axillary ultrasonography followed by core needle biopsy of suspicious nodes; no. of false negatives = number of patients with normal/negative test but histologically extensive nodal involvement; no. of true negatives = number of patients with normal/negative test and histologically no/limited nodal involvement; NPV = negative predictive value; FNR = false-negative rate.

**Table 3:** Summary of studies on MRI to exclude patients with extensive nodal involvement.

Study		Year	Field strength	No. of patients	No. (%) of patients with extensive nodal involvement	No. of false negatives	No. of true negatives	NPV (%)	FNR (%)
*Studies in which extensive nodal involvement was defined as pN2-3*									
Unenhanced MRI (T1w/T2w)									
Nijnatten van et al. [27]	Reader 1	2016	1.5 T	377	16 (4.2%)	3	318	99	19
	Reader 2	2016	1.5 T	377	16 (4.2%)	2	297	99	13
DCE-MRI									
Hwang et al. [40]		2013	1.5 T	349	18 (5.2%)	4	272	99	22
Hyun et al. [41]		2016	3.0 T	310	12 (3.9%)	1	256	99.6	8

*Studies in which extensive nodal involvement was defined as ≥3 positive nodes*									
Unenhanced MRI (T1w/T2w)									
Barco et al. [16]		2016	1.5 T	1351	182 (13.5%)	100	1066	91	55
DCE-MRI									
Hieken et al. [17]		2013	1.5 T	505	52 (10.3%)	10	327	97	19
Kim et al. [18]		2019	3.0 T	256	17 (6.6%)	2	179	99	12

MRI = magnetic resonance imaging; DCE-MRI = dynamic contrast-enhanced MRI (using gadolinium-based contrast agents); No. of false negatives = number of patients with normal/negative MRI but histologically extensive nodal involvement; no. of true negatives = number of patients with normal/negative MRI and histologically no/limited nodal involvement; NPV = negative predictive value; FNR = false-negative rate.

**Table 4:** Summary of studies on 18F-FDG PET/CT to exclude patients with extensive nodal involvement.

Study	Year	No. of patients	No. (%) of patients with extensive nodal involvement	No. of false negatives	No. of true negatives	NPV (%)	FNR (%)
*Study in which extensive nodal involvement was defined as pN2-3*							
Hwang et al. [40]	2013	349	18 (5.2%)	5	288	98	28

*Study in which extensive nodal involvement was defined as ≥3 positive nodes*							
Ahn et al. [32]	2017	364	43 (11.8%)	9	250	97	21

No. of false negatives = number of patients with normal/negative PET/CT but histologically extensive nodal involvement; no. of true negatives = number of patients with normal/negative PET/CT and histologically no/limited nodal involvement; NPV = negative predictive value; FNR = false-negative rate.

**Table 5:** Summary of accuracy of preoperative axillary imaging modalities to exclude patients with ≥3 positive nodes.

Staging modality	No. of studies	Total no. of patients	Prevalence of extensive nodal involvement (mean)	NPV (mean)	FNR (mean)
AxUS	8	12.143	6.1–18.1 (12.1)	92–98 (95)	30–37 (34)
US/FNAC	6	6.078	4.7–18.9 (11.1)	93–97 (95)	15–58 (43)
MRI	3	2.112	6.6–13.5 (11.9%)	91–99 (93)	12–55 (45)
Unenhanced MRI	1	1.351	13.5	91	55
DCE-MRI	2	761	6.6–10.3 (9.1)	97–99 (98)	12–19 (17)
PET/CT	1	364	11.8	97	21

No. = number; FNR = false-negative rate; NPV = Negative Predictive Value; AxUS = Axillary Ultrasonography; US/FNAC = AxUS followed by fine-needle aspiration cytology of suspicious node; MRI = Magnetic Resonance Imaging; DCE = Dynamic Contrast Enhanced; PET = Positron Emission Tomography; PET/CT = combined PET and Computed Tomography.
